# Electrocardiographic Imaging Using a Spatio-Temporal Basis of Body Surface Potentials—Application to Atrial Ectopic Activity

**DOI:** 10.3389/fphys.2018.01126

**Published:** 2018-08-22

**Authors:** Steffen Schuler, Andreas Wachter, Olaf Dössel

**Affiliations:** Institute of Biomedical Engineering, Karlsruhe Institute of Technology, Karlsruhe, Germany

**Keywords:** ECG, inverse problem, spatio-temporal regularization, basis vectors, body surface potentials, atrial ectopic beats

## Abstract

Electrocardiographic imaging (ECGI) strongly relies on a priori assumptions and additional information to overcome ill-posedness. The major challenge of obtaining good reconstructions consists in finding ways to add information that effectively restricts the solution space without violating properties of the sought solution. In this work, we attempt to address this problem by constructing a spatio-temporal basis of body surface potentials (BSP) from simulations of many focal excitations. Measured BSPs are projected onto this basis and reconstructions are expressed as linear combinations of corresponding transmembrane voltage (TMV) basis vectors. The novel method was applied to simulations of 100 atrial ectopic foci with three different conduction velocities. Three signal-to-noise ratios (SNR) and bases of six different temporal lengths were considered. Reconstruction quality was evaluated using the spatial correlation coefficient of TMVs as well as estimated local activation times (LAT). The focus localization error was assessed by computing the geodesic distance between true and reconstructed foci. Compared with an optimally parameterized Tikhonov-Greensite method, the BSP basis reconstruction increased the mean TMV correlation by up to 22, 24, and 32% for an SNR of 40, 20, and 0 dB, respectively. Mean LAT correlation could be improved by up to 5, 7, and 19% for the three SNRs. For 0 dB, the average localization error could be halved from 15.8 to 7.9 mm. For the largest basis length, the localization error was always below 34 mm. In conclusion, the new method improved reconstructions of atrial ectopic activity especially for low SNRs. Localization of ectopic foci turned out to be more robust and more accurate. Preliminary experiments indicate that the basis generalizes to some extent from the training data and may even be applied for reconstruction of non-ectopic activity.

## 1. Introduction

Reconstructing the heart's electrical activity from non-invasively measured body surface potentials (BSP) is known as the inverse problem of electrocardiography (Pullan et al., 2010). The ill-posedness of this problem can be overcome by introducing additional information—a technique called regularization. Classical regularization methods such as Tikhonov regularization add the “information” that the solution must be of small signal energy or smooth in space or time. However, they do not complement the measurements with physiological information about the spread of cardiac excitation, which could greatly improve the uniqueness of the solution. For example, ambiguities can arise between sources on two different regions oriented in parallel, when body surface potentials are projected back onto the heart. Incorporating information about the spatio-temporal coherence of excitation spread, i.e., taking into account that excitation waves can only gradually propagate across connected regions, is expected to help resolve ambiguities. Several approaches have been proposed to incorporate electrophysiological knowledge (van Oosterom, 1999; Messnarz et al., 2004; Ghodrati et al., 2006; Wang et al., 2010; Potyagaylo et al., 2014, 2016a; Cluitmans et al., 2018). Cluitmans et al. (2017) reconstructed potentials on the ventricular epicardium as sparse combinations of spatial source basis vectors generated from simulations of many paced beats. In this work, we use a related approach. However, we suggest to create a basis of body surface potential patterns instead of source patterns to condense the information to what can possibly be measured on the body surface. Corresponding basis patterns in source space are then obtained and combined to express reconstructions. Furthermore, we use a spatio-temporal basis instead of a spatial-only one, as we believe this reduces ambiguities and increases the robustness to noise. We demonstrate the method in an application to atrial ectopic activity.

## 2. Methods

**Figure 1:**
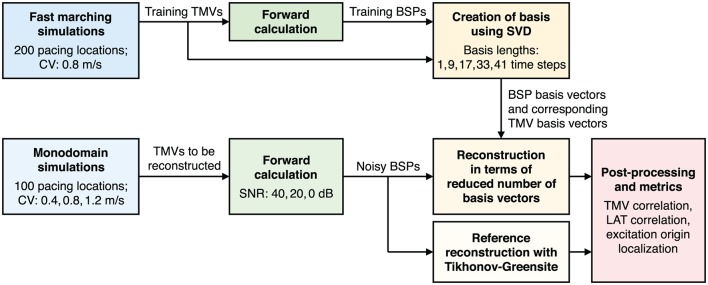
Flowchart illustrating the outline of this study.

The outline of this study is illustrated in Figure 1. First, fast marching simulations of 200 ectopic foci are performed and forward calculated to create basis vectors. Reconstructions are then performed using these basis vectors for another set of 100 ectopic foci simulations. Here, the monodomain model is used with three different conduction velocities (CV) and forward calculated BSPs are corrupted with three different levels of noise. Finally, reference reconstructions are obtained using the Tikhonov-Greensite method and the same metrics are calculated for both reconstruction methods.

### 2.1. Geometries

**Figure 2:**
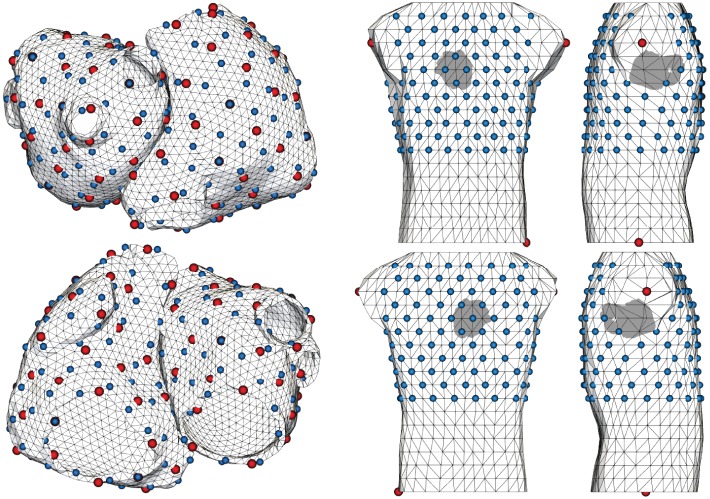
Meshes of the atria and torso used for forward and inverse calculations. Blue spheres on the atria indicate 200 pacing locations used to create basis vectors, while red spheres indicate 100 pacing locations used for monodomain simulations to be reconstructed. Torso electrodes are shown in blue. Red spheres on the torso mark reference nodes.

Figure 2 shows the geometries used for forward and inverse calculations. They are the same as in Schuler et al. (2017), which in turn are based on Figuera et al. (2016). The surface meshes of the atria and torso consist of 4,800 and 844 nodes and have an average edge length of 3.4 and 27.0 mm, respectively. A subset of 173 torso nodes have been selected as electrodes (blue spheres). Much finer tetrahedral meshes of the atria were used for excitation simulations (142 k nodes, average edge length: 0.9 mm). Blue and red spheres on the atria mark evenly distributed pacing locations used for fast marching and monodomain simulations, respectively. Note that pacing locations for both sets of simulations do generally not coincide.

### 2.2. Fast marching simulations

For creating basis vectors, fast marching simulations of 200 paced beats were performed. First, local activation times (LAT) *t*_*a*_ were computed by solving the eikonal equation with the fast marching method (Pernod et al., 2011):

$$\begin{array}{l} ||\nabla t_{a}|{|}_{2}=\frac{1}{c} \end{array}$$

**Figure 3:**
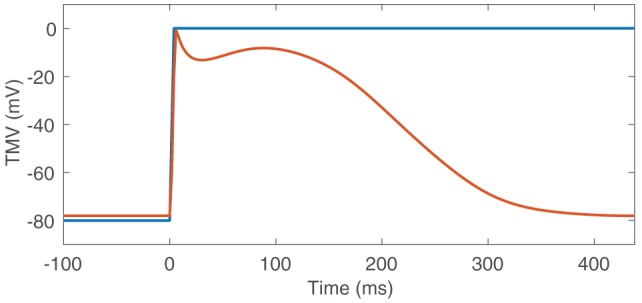
TMV templates based on the Courtemanche et al. cell model (red) and the step function (blue).

The CV was homogeneously set to *c* = 0.8m/s. A TMV template was then aligned with LATs. Experiments with a template based on the Courtemanche et al. cell model and a step-function-like template (Figure 3) showed that basis vectors created as described in section 2.5.1 are mainly determined by the depolarization upstroke and it is not necessary to include the repolarization. Therefore, we decided to use the step-function-like template. The temporal sampling period was chosen to be 2ms.

### 2.3. Monodomain simulations

As activity to be reconstructed, 100 paced beats were simulated using the monodomain model:

$$\begin{array}{l} \nabla \cdot \left(\sigma_{mono}\nabla V_{m}\right)=\beta \left(C_{m}\frac{\partial V_{m}}{\partial t}+I_{ion}\right) \end{array}$$

The monodomain conductivity σ_*mono*_ was chosen homogeneous and isotropic and, together with the surface-to-volume ratio β, was adjusted to obtain the desired CV. In order to study what happens, if a wrong CV is assumed for the creation of basis vectors, we varied the CV by ±50% of its baseline value. Therefore, three different CVs were used for monodomain simulations: 0.4, 0.8, and 1.2m/s. Ionic currents *I*_*ion*_ across the cell membrane were defined according to Courtemanche et al. (1998). As for fast marching simulations, the temporal resolution is 2ms.

### 2.4. Forward calculation

According to bidomain theory, extracellular potentials ϕ are related to volumetric TMVs *V*_*m*_ by:

(1) $$\begin{array}{l} \nabla \cdot \left(\left(\sigma_{i}+\sigma_{e}\right)\nabla \phi \right)=-\nabla \cdot \left(\sigma_{i}\nabla V_{m}\right) \end{array}$$

In this work, we assume isotropic, i.e., scalar intra- and extra-cellular conductivities **σ**_*i*_ and **σ**_*e*_, respectively. As this is a special case of equal intra- and extra-cellular anisotropy ratios, volumetric TMVs may be replaced by TMVs on the myocardial surface (Yamashita and Geselowitz, 1985). For a homogeneous torso with the same bulk conductivity as the heart σ, the potential ϕ at an observation point can be calculated from surface TMVs *V*_*m*_ by (Simms and Geselowitz, 1995):

(2) $$\begin{array}{l} \phi =-\frac{1}{4\pi \sigma }\int_{S_{H}}\sigma_{i}V_{m}\;d\Omega_{H}\;-\frac{1}{4\pi }\int_{S_{B}}\phi_{B}\;d\Omega_{B}\; \end{array}$$

*S*_*H*_ and *S*_*B*_ are the surfaces bounding the myocardium and torso, respectively. *dΩ*_*H*_ and *dΩ*_*B*_ are the solid angles subtended by an area element on the corresponding surface as seen from an observation point. By applying the boundary element method to solve (2) for BSPs ϕ = ϕ_*B*_, a lead field matrix **A** is obtained that transforms surface TMVs to BSPs. Intracellular and bulk conductivities are set to σ_*i*_ = 0.05S/m, and σ = 0.2S/m and potentials are referenced to Wilson's central terminal. We assume the following linear forward model:

$$\begin{array}{l} {\text{b}}_{k}=\text{A}{\text{x}}_{k}+\varepsilon_{k} \end{array}$$

where **b**_*k*_, **x**_*k*_ and **ε**_*k*_ are BSPs, TMVs, and white Gaussian noise for all nodes at a time step *k*, respectively. For fast marching simulations used to create basis vectors, we do not add any noise. For monodomain simulations, we consider three different signal-to-noise ratios (SNRs): 40, 20, and 0dB. It is assumed that each electrode is affected by the same absolute noise power, which is set to the average signal power of all electrodes divided by the SNR. For solving the inverse problem, we assume perfect knowledge of **A** and thus neglect errors due to imperfect geometries and conductivities.

### 2.5. Reconstruction using spatio-temporal BSP basis

The following identifiers and terminology will be used in this section:

**Table:** 

*N*	Number of atria nodes
*M*	Number of torso electrodes
*K*	Total number of time steps in training data (here: 200) or to be reconstructed
*L*	Basis length: odd number of time steps in each basis vector (here: 1, 9, 17, 25, 33, 41)
*P*	Basis dimension: number of basis vectors used for reconstruction

#### 2.5.1. Creation of basis vectors

As in Cluitmans et al. (2017), we use the singular value decomposition (SVD) to create basis vectors. In order to get a spatio-temporal basis, we define an observation as the column-wise concatenation of values at all nodes for all time steps within a time window of length *L*. As we do not know the time delay between the activity to be reconstructed and the activities used to create the basis, we include all possible delays by continuously time-shifting the window by a single time step. This way, a total of *K*−*L*+1 observations are generated for each simulation. Row-wise concatenation of all observations (all time shifts of all simulations) then yields a data matrix **D**. Using **x**_*k*_ to denote TMVs for all nodes at a time step *k*, the TMV data matrix **D**_**x**_ is thus given as:

$${\text{D}}_{\text{x}}=\left[\begin{array}{c} \left(\begin{array}{cccc} {\text{x}}_{1}^{⊤} & {\text{x}}_{2}^{⊤} & \cdots & {\text{x}}_{L}^{⊤}\\ {\text{x}}_{2}^{⊤} & {\text{x}}_{3}^{⊤} & \cdots & {\text{x}}_{L+1}^{⊤}\\ \vdots & \vdots & \; & \vdots \\ {\text{x}}_{K-L+1}^{⊤} & {\text{x}}_{K-L+2}^{⊤} & \cdots & {\text{x}}_{K}^{⊤} \end{array}\right)\\ \vdots \\ \left(\text{repeat for all simulations}\right) \end{array}\right]$$

By replacing **x** with **b**, a BSP data matrix **D**_**b**_ can be constructed in exactly the same way. From **Ax**_*k*_ = **b**_*k*_, it follows that the whole TMV data matrix can be forward calculated using a block diagonal lead field matrix $\tilde{A}$:

(3) $$\begin{array}{l} \tilde{A}{\text{D}}_{\text{x}}^{⊤}={\text{D}}_{\text{b}}^{⊤}\;\Leftrightarrow \;{\text{D}}_{\text{b}}={\text{D}}_{\text{x}}{\tilde{A}}^{⊤}\;\text{with }\tilde{A}={\text{I}}_{L}⊗\text{A} \end{array}$$

**I**_*L*_ is the *L*×*L* identity matrix and ⊗ denotes the Kronecker product. We now perform an SVD of the BSP data matrix:

(4) $$\begin{array}{l} {\text{D}}_{\text{b}}=\text{U}\text{S}{\text{V}}_{\text{b}}^{⊤} \end{array}$$

The columns of **V**_**b**_ are spatio-temporal basis vectors of BSPs. We now want to find the corresponding TMV basis vectors **V**_**x**_, for which holds:

(5) $$\begin{array}{l} {\text{V}}_{\text{b}}=\tilde{A}{\text{V}}_{\text{x}} \end{array}$$

Substituting (3) and (5) in (4) yields:

$$\begin{array}{l} {\text{D}}_{\text{x}}{\tilde{A}}^{⊤}=\text{U}\text{S}{\text{V}}_{\text{x}}^{⊤}{\tilde{A}}^{⊤}\;\Leftrightarrow \;{\text{V}}_{\text{x}}^{⊤}={\left(\text{U}\text{S}\right)}^{+}{\text{D}}_{\text{x}}={\text{S}}^{+}{\text{U}}^{⊤}{\text{D}}_{\text{x}} \end{array}$$

(·)^+^ denotes the Moore–Penrose pseudoinverse. This shows that the TMV basis can directly be calculated from the TMV data matrix using an inversion of **US**, the scores matrix obtained from the SVD of the BSP data matrix. An inversion of $\tilde{A}$ is not required.

#### 2.5.2. Reconstruction in terms of basis vectors

Now the BSPs of a patient show up. They will be called **B** = [**b**_1_, **b**_2_, …, **b**_*K*_] in the following. Here, *K* is the total number of time steps to be reconstructed. To reconstruct TMVs in terms of a reduced number *P* of basis vectors, we first perform a least-squares regression using BSP basis vectors. This results in the optimal basis vector weights **W**:

(6) $$\begin{array}{l} \text{W}=\arg\underset{\text{W}}{\min}||{\text{V}}_{\text{b}}\left(:,1:P\right)\text{W}-\tilde{B}{\|}_{F}^{2} \end{array}$$

||·||_*F*_ denotes the Frobenius norm and $\tilde{B}$ are the “measured” BSPs reshaped into the format of basis vectors. Using MATLAB notation, they are given by:

$$\begin{array}{l} \tilde{B}\left(:,k\right)=\text{reshape}\left(\text{B}\left(:,k:k+L-1\right),LM,1\right)\;\text{for }k=1,2,\ldots ,\\ \;K-L+1 \end{array}$$

Since the columns of **V**_**b**_ form an orthonormal basis, the solution to (6) is given by:

(7) $$\begin{array}{l} \text{W}={\text{V}}_{\text{b}}{\left(:,1:P\right)}^{⊤}\;\tilde{B} \end{array}$$

This can be seen as filtering the BSPs by projecting them onto the *P* most important BSP basis vectors. The weights **W** are now used to obtain the reconstructed TMVs ${\tilde{X}}_{r}$ as linear combination of corresponding TMV basis vectors:

$$\begin{array}{l} {\tilde{X}}_{r}={\text{V}}_{\text{x}}\left(:,1:P\right)\text{W} \end{array}$$

Each column of ${\tilde{X}}_{r}$ contains the row-wise concatenation of all time windows with length *L*. As final solution **X**_*r*_, we therefore extract the central time step of each window:

$$\begin{array}{l} {\text{X}}_{r}={\text{V}}_{\text{x}}\left(\frac{L-1}{2}N+\left(1:N\right),1\text{:}P\right)\text{W} \end{array}$$

Corresponding BSPs can be obtained in the same manner:

$$\begin{array}{l} {\text{B}}_{r}={\text{V}}_{\text{b}}\left(\frac{L-1}{2}M+\left(1:M\right),1\text{:}P\right)\text{W}=\text{A}{\text{X}}_{r} \end{array}$$

We would like to point out that creating the basis vectors in BSP space instead of in source space, i.e., calculating the SVD of **D**_**b**_ instead of **D**_**x**_, is the key step which makes it possible to perform an unregularized least-squares regression without additional constraints. If the basis vectors were created in source space, they may still contain redundant information with respect to BSPs and therefore a regularized regression would be necessary: $\text{W}=\arg\underset{\text{W}}{\min}\left\{{\left\|\tilde{A}{\text{V}}_{\text{x}}\left(:,1:P\right)\text{W}-\tilde{B}\right\|}_{F}^{2}+\lambda {\left\|\text{R}{\text{V}}_{\text{x}}\left(:,1:P\right)\text{W}\right\|}_{F}^{2}\right\}$, where **R** is a regularization matrix. This would require inverting a *P* × *P* matrix for every combination (*P*, λ), while only a fast matrix multiplication (7) has to be computed for each *P* when using the BSP basis.

#### 2.5.3. Choice of basis dimension

**Figure 4:**
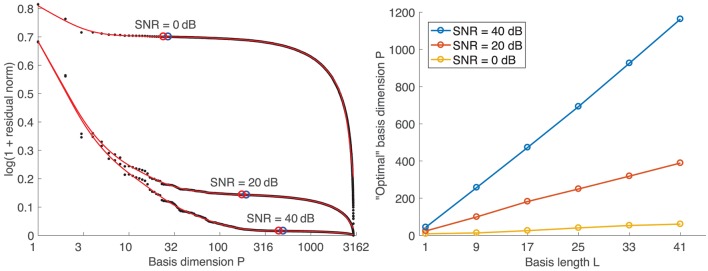
**(Left)** Exemplary “L-curves” for a basis length of *L* = 17. Black dots: original data points. Red curves: smoothing spline fits. Blue circles: Points of min. absolute slope. Red circles: Points 10% left of min. slope points. **(Right)** Dependency of basis dimension on basis length.

Following the concept of the L-curve, the “optimal” basis dimension is determined from the log-log graph of the residual norm ||**B**_*r*_−**B**||_*F*_ versus the corresponding basis dimension *P* (left diagram in Figure 4). Instead of the maximal curvature, which is not very pronounced in the resulting “L-curves,” we found that the minimal absolute slope (blue circles) of a smoothing spline fit is a good criterion for selecting the basis dimension. To ensure that solutions are not underregularized, we set the basis dimension 10% lower than at the point of minimal absolute slope (red circles). The right diagram in Figure 4 shows that the resulting basis dimension for one specific SNR depends linearly on the basis length *L*.

### 2.6. Reference reconstruction with tikhonov-greensite

For comparison, reconstructions with the Tikhonov-Greensite method (TikhGS) are performed (Greensite and Huiskamp, 1998). While standard Tikhonov methods regularize each time step individually, TikhGS performs Tikhonov regularization for the *p* most important temporal singular vectors of BSPs, which helps to eliminate noise and generally leads to better reconstructions, given the number of singular vectors used is chosen appropriately. We apply the epi-endo projection described in Schuler et al. (2017), which further improves the solution. TikhGS is then used with simultaneous zero- and second-order constraints:

$$\begin{array}{l} \;B\;=USV^{⊤},\;\bar{V}=V(:,1:p)\\ X\bar{V}=\arg\underset{\left(X\bar{V}\right)}{\min}\left\{\|A(X\bar{V})-B\bar{V}{\|}_{F}^{2}+\text{λ}\|L(X\bar{V}){\|}_{F}^{2}+\eta \|X\bar{V}{\|}_{F}^{2}\right\}\\ \;X\;=(X\bar{V}){\bar{V}}^{⊤} \end{array}$$

In order to provide the reference method with the best possible parameters, *p* was varied between 3 and 13 and regularization parameters λ and η were optimized for each case individually to maximize the mean of spatial TMV correlation with the ground truth over the period of depolarization. The downhill simplex method was used for optimization.

### 2.7. Post-processing and metrics

#### 2.7.1. Transmembrane voltages

To assess the quality of reconstructed TMVs, the Pearson correlation coefficient between reconstructions and the ground truth is computed separately for each time step across all nodes (spatial CC). As we want to quantify, how good the depolarization is being reconstructed, we only calculate the spatial CC for time steps between the first and the last activation of each simulation, as defined by the ground truth.

#### 2.7.2. Local activation times

**Figure 5:**
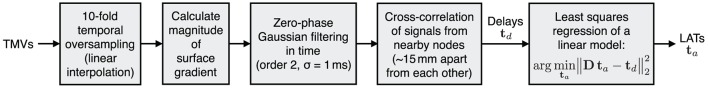
Delay-based LAT estimation. **D** is a difference matrix to obtain pair-wise delays from LATs.

Local activation times represent one of the most important characteristic of cardiac excitation spread and are therefore estimated from reconstructed TMVs. We use the “global activation time” approach described in Dubois et al. (2012), which is based on cross-correlating signals of nearby nodes to find their time delay. The method has further been advanced in Duchateau et al. (2017) to combine delay-based and deflection-based activation times. In this work, however, we stick with the delay-only formulation. The procedure is illustrated in Figure 5. First, TMVs are oversampled to allow for a precise alignment in time. As signal for cross-correlation, we then use a Gaussian filtered version of the magnitude of the surface gradient of TMVs: ||∇*V*_*m*_(*t*)||_2_. Using a lowpass filtered time derivative as described in Duchateau et al. (2017) yielded unsatisfactory results for both TikhGS and the BSP basis reconstruction. This might be explained by the fact that the spatial gradient of TMVs is the source of body surface potentials according to (1). LATs are finally estimated from the delays using least-squares regression of a linear model. As LAT metric, we calculate the spatial CC between LATs estimated for reconstructions and the ground truth.

#### 2.7.3. Ectopic focus localization

**Figure 6:**
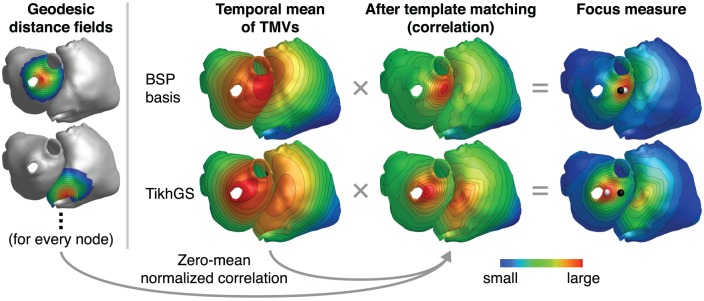
Ectopic focus localization illustrated for exemplary reconstructions with the BSP basis (*L* = 33) and TikhGS. CV = 0.8m/s. SNR = 0dB. Black spheres: true focus, white spheres: reconstructed focus. Geodesic distance fields shown on the left are reversed and truncated at 3 cm (dark blue). Gray areas are not taken into account for correlation.

BSPs are directly proportional to the solid angle at a measurement point subtended by the depolarization wavefront, which separates regions of low and high TMVs (see Equation 2). For a given noise level, the instantaneous SNR therefore rises with the size of the depolarization wavefront and the excitation origin cannot reliably be determined from the small signal at the very onset of excitation. If the depolarization wavefront spreads too far from the origin, however, the uncertainty of localizing the origin within the depolarized region increases as well. As the CV is not known beforehand, we therefore do not base the focus localization on one specific time step, but use the temporal mean of many time steps after excitation onset. If the TMV waveform was a Heaviside step function, the TMV time integral (and thus also its temporal mean) would be proportional to reversed activation times. For other TMV waveforms, the temporal mean still yields a valid activation “sequence,” as long as the TMV time integral is increasing (Schulze, 2015). Based on the TMV waveform of the Courtemanche et. al. model (Figure 3), we chose to calculate the mean over 200 ms after excitation onset. In general, this time should not be chosen larger than the effective refractory period. In this work, the time of excitation onset is assumed to be known. In practice, it would have to be determined as the P wave onset. Having obtained activation sequences, a template matching approach is employed to detect the focus location: For every mesh node, the zero-mean normalized correlation (ZNC) of the temporal mean of TMVs $\bar{V_{m}}$ and the reversed geodesic distance field, originating from the respective node and truncated at 3 cm, is calculated. As the ZNC only measures similarity in shape, not magnitude, the result is further weighted with $\left(\bar{V_{m}}-\min\left\{\bar{V_{m}}\right\}\right)$. The maximum of this “focus measure” is finally detected as focus. The method is illustrated in Figure 6 for both BSP basis and TikhGS reconstructions. Correlating with the geodesic distance fields can also be seen as a transformation to find the center of mass of the TMV distribution on a curved surface.

As localization error, the geodesic distance between the true and reconstructed focus is evaluated. In contrast to the Euclidean distance, this metric correctly yields large errors for nearby points that are not directly connected via the geometry, such as two points on the opposite side of the interatrial region.

## 3. Results

### 3.1. Basis vectors and singular values

**Figure 7:**
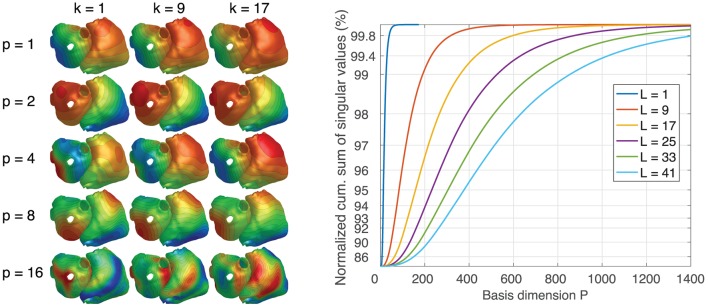
**(Left)** Examples of TMV basis vectors for *L* = 17. *p*: basis vector number. *k*: time step. Colors only visualize spatial morphology. **(Right)** Cumulative sum of singular values for different basis lengths *L*.

Figure 7 (left) depicts 3 time steps of exemplary TMV basis vectors for a basis length of *L* = 17. It can be seen that the spatial and/or temporal frequency increases with the basis vector number and that spatial patterns evolve over time. The diagram on the right shows that the larger the basis length, the more basis vectors are needed to represent the same proportion of information contained within all basis vectors.

### 3.2. Robustness to noise

**Figure 8:**
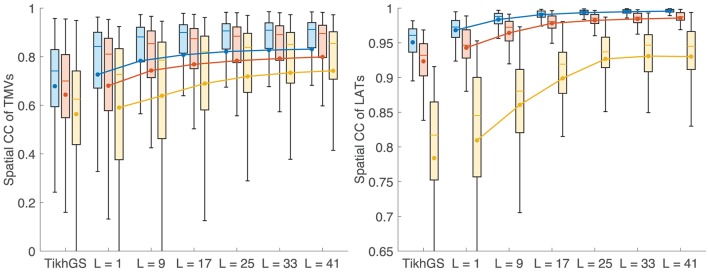
Metrics for a CV of 0.8m/s. Colors represent different SNRs. Blue: 40dB, red: 20dB, yellow: 0dB. Boxes: 25–75th percentile. Whiskers: 1.5 inter-quartile range. Filled circles and lines represent the mean. **(Left)** Spatial CC of TMVs. **(Right)** Spatial CC of LATs.

**Figure 9:**
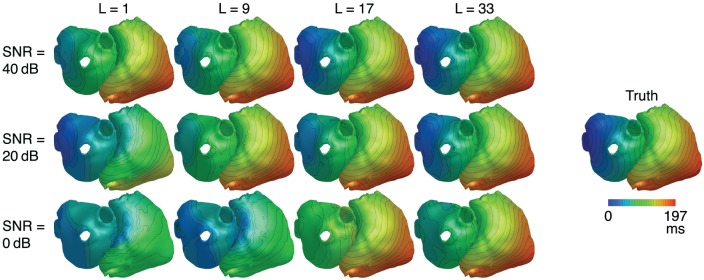
LATs for different SNRs and basis lengths *L*. CV = 0.8m/s.

Metrics for reconstructions of monodomain simulations with the same CV as used for basis creation are shown in Figure 8. For all noise levels considered, BSP basis reconstructions perform consistently better than TikhGS. Even a spatial-only basis (*L* = 1) leads to an improvement. Increasing the basis length further increases the correlation coefficients for both TMVs and LATs, especially for low SNRs. This can also be seen from Figure 9, where LATs for an ectopic focus near the left inferior pulmonary vein are shown for different SNRs and basis lengths. While the LAT map for *L* = 1 and 40dB already looks much like the ground truth, basis lengths of at least *L* = 9 and *L* = 17 are needed for 20 and 0dB, respectively.

### 3.3. Influence of conduction velocity

**Figure 10:**
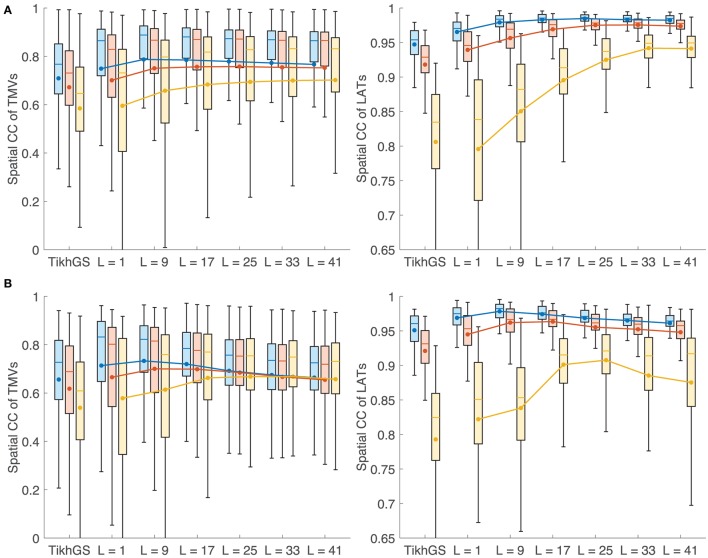
**(A)** Metrics for a CV of 0.4m/s. **(B)** Metrics for a CV of 1.2m/s. See caption of Figure 8 for details.

**Figure 11:**
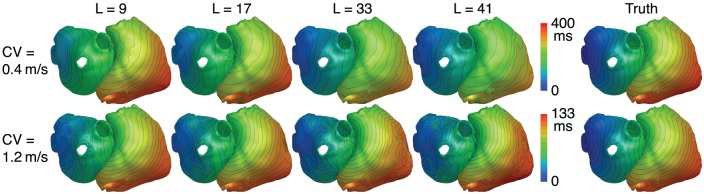
LATs for different CVs and basis lengths *L*. SNR = 40dB.

In theory, errors in the spatial dimension, i.e., the position of the wavefront, increase linearly with time for a mismatch of CV. Therefore it is expected that the reconstruction quality deteriorates for large basis lengths if the CV used to create basis vectors deviates from the actual CV. Figures 10A,B show the metrics for a CV of 0.4 and 1.2m/s, respectively, while the CV assumed for basis vectors remains at 0.8m/s. It can be seen that there is now indeed an upper limit for the improvement with increasing basis lengths. Results show a clear tradeoff between the error due to a wrong CV for large basis lengths and the error due to lower robustness to noise for small basis lengths. Although the BSP basis reconstruction still outperforms TikhGS in every case, it can be seen from comparison of both figures that overestimating the true CV during basis creation seems to be less problematic than underestimating it. Figure 11 shows another interesting effect of wrong CVs. If the CV is overestimated (top row), LATs estimated from reconstructions with large basis lengths are smaller than true LATs, suggesting a larger than actual CV. To a lesser extent, the opposite effect can be seen for an underestimation (bottom row).

### 3.4. Ectopic focus localization

**Figure 12:**
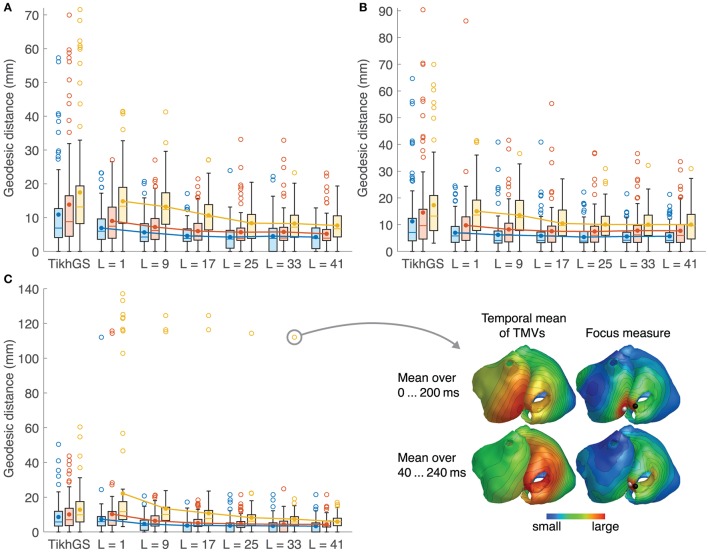
Localization errors for all 100 ectopic foci. See caption of Figure 8 for colors and markers. Outliers are shown as empty circles. **(A)** CV = 0.8m/s. **(B)** 1.2m/s. **(C)** 0.4m/s. The outlier for CV = 0.4m/s and *L* = 33 is depicted on the bottom right. Black sphere: true focus, white sphere: reconstructed focus.

Localization errors are shown in Figure 12. The mean error decreases with increasing basis length, even for CVs different from 0.8m/s. For *L* = 41, it is approximately halved compared to TikhGS (see Table 1). Another important improvement is the reduction of the maximum localization error. While errors for TikhGS range up to 90mm, the maximum error for *L* = 41 is 34mm. For a CV of 0.4m/s, however, there is an outlier for *L* = 25 and *L* = 33. This case is illustrated on the right of Figure 12C. For this ectopic focus at the orifice of the inferior vena cava, the reconstruction first shows a false activity on the nearby left atrium. Only after the excitation has further increased in size, the reconstruction continues to show the activity at the correct location. A better localization thus would have been obtained for a later (or longer) time window used for calculating the temporal mean.

**Table 1:**
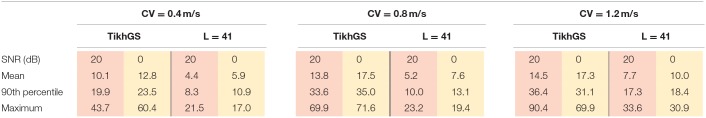
Localization errors in mm.

**Figure 13:**
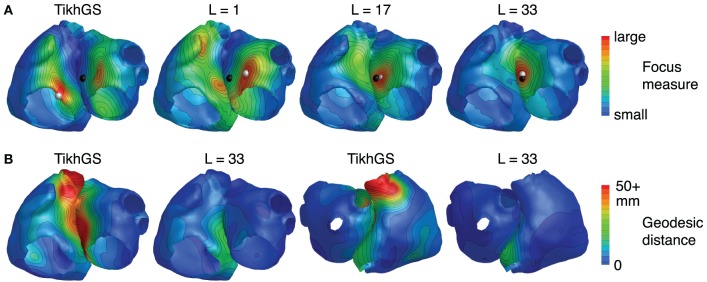
**(A)** Focus measure for a focus on the anterior-septal wall of the left atrium (CV = 0.8m/s, SNR = 0dB). Black sphere: true focus, white sphere: reconstructed focus. **(B)** Distribution of localization errors across the atria for TikhGS and the BSP basis reconstruction (CV = 0.8m/s, SNR = 20dB, results for 0dB are qualitatively similar). Values were interpolated by minimizing the Laplacian at all nodes (Oostendorp et al., 1989).

In general, the BSP basis reconstruction largely resolves ambiguities. In Figure 13A, this is demonstrated for a focus on the anterior-septal wall of the left atrium. TikhGS and a purely spatial basis fail to recover the activity at the correct spot and show multiple activities on the right and left atrium instead. Using a spatio-temporal basis, however, leads to a reconstruction at the correct spot. Increasing the basis length progressively increases the uniqueness of the solution. An even better impression on where ambiguities arise between sources can be obtained by taking a look at the distributions of localization errors across the atria, as shown in Figure 13B. For TikhGS, the largest errors occur at the interatrial region, where left and right atrial surfaces are very close to each other and oriented in parallel. Using a BSP basis of sufficient length greatly reduces these errors.

### 3.5. Effect of non-conducting region

**Figure 14:**
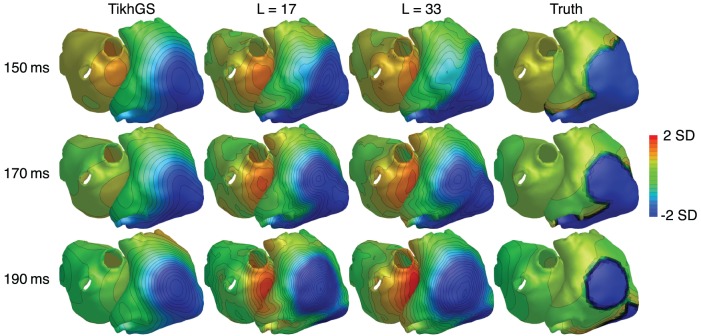
TMVs for a simulation containing a non-conducting scar region on the right atrium. SNR = 20dB. CV = 0.8m/s. Each time step was normalized by subtracting the spatial mean and dividing by the spatial standard deviation (SD).

In order to test, how much the reconstruction relies on the excitation patterns in the training data, we added a non-conducting scar region to one simulation. For that purpose, the monodomain conductivity was set to zero in a circular region with a diameter of 4cm on the right atrium. The results are shown in Figure 14. It can be seen that the reconstructed wavefront propagates around the non-conducting region on the right atrium, even though the training data did not include such a pattern. The basis created by including all time shifts of excitation patterns in the data matrix therefore generalizes from individual patterns.

## 4. Discussion

We demonstrated that using a spatio-temporal basis of BSPs to reconstruct TMVs improves the robustness to noise, resolves ambiguities between sources and leads to better localization of atrial ectopic foci than TikhGS. Of all possible solutions fitting to measured BSPs, the proposed method selects the one which is most probable with regard to the training data used to create basis vectors. This approach allows us to reconstruct hidden sources as well, given they occur in conjunction with other, visible sources. Compared to simply correlating measured BSPs with BSPs of many simulated beats (Potyagaylo et al., 2016b), the new method has two main advantages: First, the basis generalizes to some extent from the individual activities used as input and thus also allows us to represent excitation patterns that were not among the training data. Second, measured and simulated BSPs do not have to be aligned in time, as the basis contains all time shifts. Localization results obtained by correlating with all BSP patterns in the training data and finding the one with the maximum correlation coefficient are included in the Supplementary Material. Although this approach works comparably well if the CV matches the CV in the training data, it performs considerably worse than the BSP basis reconstruction for non-matching CVs.

One remaining question regarding the creation of basis vectors is how to best set up the training data, so that each basic activation pattern occurs equally often in the data matrix. In the current geometry, the left and right atrium are only connected by one bridge representing the Bachmann's bundle. This results in almost the same activation pattern on the opposite atrium, once the excitation has passed the bridge. This activation pattern (corresponding to pacing at the bridge) occurs disproportionately often in the data matrix and will therefore best be represented by the resulting basis vectors. Reconstructions might therefore be biased toward this specific pattern. In geometries with multiple bridges, this effect is not as pronounced. Limiting the information in the data matrix to a time window around the beginning of excitations is expected to optimize the basis for better reconstructions near the excitation origin.

### 4.1. Limitations

There are several limitations of this study that need to be acknowledged. The most important one is that atrial conductivities were assumed to be homogeneous and isotropic. Although we think this is a logical first step in evaluating the new reconstruction method systematically, its performance has to be studied with atrial anisotropy and several atrial geometries. This has to be done in a future work. However, exemplary reconstructions for four ectopic foci in a highly anisotropic model of the atria are included in the Supplementary Material. These results indicate that the method improves reconstructions over TikhGS for anisotropic spread of excitation as well, although the reconstruction quality does decrease compared to the isotropic case. Another aspect not considered is fibrosis, which may hamper the localization of atrial ectopic foci (Godoy et al., 2018). Finally, further studies are needed to evaluate the sensitivity to imperfect geometries and conductivities.

### 4.2. Outlook

We are planning to study the new reconstruction method with clinical measurements during (focal) ventricular tachycardia. We hope to make use of scar-related information from late gadolinium enhancement MRI during the creation of basis vectors. For non-focal arrhythmias, it would be interesting to see whether including non-focal activities in the basis is beneficial.

## Author contributions

SS developed the BSP basis reconstruction method, designed and conducted the simulation study and wrote the manuscript. AW created atrial fiber orientations for the simulations in the Supplementary Material. OD provided technical expertise and contributed during study design and manuscript preparation.

### Conflict of interest statement

The authors declare that the research was conducted in the absence of any commercial or financial relationships that could be construed as a potential conflict of interest.

## Supplementary material

Movies of atrial ectopic foci reconstructions and exemplary reconstructions of rotational activity (simple atrial fibrillation from Figuera et al., 2016) can be found in the Supplementary Material.

## References

[B1] Cluitmans M. Karel J. Bonizzi P. Volders P. Westra R. Peeters R. (2018). Wavelet-promoted sparsity for non-invasive reconstruction of electrical activity of the heart. Med. Biol. Eng. Comput. [Epub ahead of print]. 10.1007/s11517-018-1831-2. 29752679 PMC6208718

[B2] Cluitmans M. J. M. Clerx M. Vandersickel N. Peeters R. L. M. Volders P. G. A. Westra R. L. (2017). Physiology-based regularization of the electrocardiographic inverse problem. Med. Biol. Eng. Comput. 55, 1353–1365. 10.1007/s11517-016-1595-5 27873155 PMC5544815

[B3] Courtemanche M. Ramirez R. J. Nattel S. (1998). Ionic mechanisms underlying human atrial action potential properties: insights from a mathematical model. Am. J. Physiol. 275(1 Pt 2), H301–H321. 9688927 10.1152/ajpheart.1998.275.1.H301

[B4] Dubois R. Labarthe S. Coudière Y. Hocini M. Haïssaguerre M. (2012). Global and directional activation maps for cardiac mapping in electrophysiology, in Computing in Cardiology (Kraków), 349–352.

[B5] Duchateau J. Potse M. Dubois R. (2017). Spatially coherent activation maps for electrocardiographic imaging. IEEE Trans. Biomed. Eng. 64, 1149–1156. 10.1109/TBME.2016.2593003 27448338

[B6] Figuera C. Suárez-Gutiérrez V. Hernández-Romero I. Rodrigo M. Liberos A. Atienza F. (2016). Regularization techniques for ECG imaging during atrial fibrillation: a computational study. Front. Physiol. 7:466 10.3389/fphys.2016.00466 27790158 PMC5064166

[B7] Ghodrati A. Brooks D. Tadmor G. MacLeod R. (2006). Wavefront-based models for inverse electrocardiography. IEEE Trans. Biomed. Eng. 53, 1821–1831. 10.1109/TBME.2006.878117 16941838

[B8] Godoy E. J. Lozano M. García-Fernández I. Ferrer-Albero A. MacLeod R. Saiz J.. (2018). Atrial fibrosis hampers non-invasive localization of atrial ectopic foci from multi-electrode signals: a 3D simulation study. Front. Physiol. 9:404. 10.3389/fphys.2018.00404 29867517 PMC5968126

[B9] Greensite F. Huiskamp G. (1998). An improved method for estimating epicardial potentials from the body surface. IEEE Trans. Biomed. Eng. 45, 98–104. 9444844 10.1109/10.650360

[B10] Messnarz B. Tilg B. Modre R. Fischer G. Hanser F. (2004). A new spatiotemporal regularization approach for reconstruction of cardiac transmembrane potential patterns. IEEE Trans. Biomed. Eng. 51, 273–281. 10.1109/TBME.2003.820394 14765700

[B11] Oostendorp T. F. van Oosterom A. Huiskamp G. (1989). Interpolation on a triangulated 3d surface. J. Comput. Phys 80, 331–343.

[B12] Pernod E. Sermesant M. Konukoglu E. Relan J. Delingette H. Ayache N. (2011). A multi-front eikonal model of cardiac electrophysiology for interactive simulation of radio-frequency ablation. Comput. Graph. 35, 431–440. 10.1016/j.cag.2011.01.008

[B13] Potyagaylo D. Cortés E. G. Schulze W. H. W. Dössel O. (2014). Binary optimization for source localization in the inverse problem of ECG. Med. Biol. Eng. Comput. 52, 717–728. 10.1007/s11517-014-1176-4 25008005

[B14] Potyagaylo D. Dossel O. van Dam P. (2016a). Influence of modeling errors on the initial estimate for nonlinear myocardial activation times imaging calculated with fastest route algorithm. IEEE Trans. Biomed. Eng. 63, 2576–2584. 10.1109/TBME.2016.2561973 27164568

[B15] Potyagaylo D. Loewe A. van Dam P. Dössel O. (2016b). ECG imaging of focal atrial excitation: evaluation in a realistic simulation setup, in Computing in Cardiology, Vol. 43 (Vancouver, BC), 113–116.

[B16] Pullan A. J. Cheng L. K. Nash M. P. Ghodrati A. MacLeod R. Brooks D. H. (2010). The inverse problem of electrocardiography, in Comprehensive Electrocardiology, eds MacFarlane P. van Oosterom A. Pahlm O. Kligfield P. Janse M. Camm J. (London: Springer), 299–344.

[B17] Schuler S. Potyagaylo D. Dssel O. (2017). ECG imaging of simulated atrial fibrillation: imposing epi-endocardial similarity facilitates the reconstruction of transmembrane voltages, in Computing in Cardiology, Vol. 44 (Rennes).

[B18] Schulze W. H. W. (2015). ECG Imaging of Ventricular Activity in Clinical Applications. Ph.D. thesis, Institute of Biomedical Engineering, Karlsruhe Institute of Technology1.

[B19] Simms H. D. J. Geselowitz D. B. (1995). Computation of heart surface potentials using the surface source model. J. Cardiovasc. Electrophysiol. 6, 522–531. 8528487 10.1111/j.1540-8167.1995.tb00425.x

[B20] van Oosterom A. (1999). The use of the spatial covariance in computing pericardial potentials. IEEE Trans. Biomed. Eng. 46, 778–787. 10396896 10.1109/10.771187

[B21] Wang L. Zhang H. Wong K. C. Liu H. Shi P. (2010). Physiological-model-constrained noninvasive reconstruction of volumetric myocardial transmembrane potentials. IEEE Trans. Biomed. Eng. 57, 296–315. 10.1109/TBME.2009.2024531 19535316

[B22] Yamashita Y. Geselowitz D. B. (1985). Source-field relationships for cardiac generators on the heart surface based on their transfer coefficients. IEEE Trans. Biomed. Eng. 32, 964–970. 4065909 10.1109/TBME.1985.325647

