# Supplementary material for: Electrocardiographic Imaging Using a Spatio-Temporal Basis of Body Surface Potentials—Application to Atrial Ectopic Activity
Source: Front Physiol. 2018 Aug 22;9:1126. doi: 10.3389/fphys.2018.01126 (PMC6129676; doi:10.3389/fphys.2018.01126)
Supplement: Supplementary file 1 [file Data_Sheet_1.pdf]

Supplementary Material: Electrocardiographic Imaging Using a Spatio-Temporal Basis of Body Surface Potentials – Application to Atrial Ectopic Activity

1 EXHAUSTIVE SEARCH CORRELATION

For comparing localization results obtained using the BSP basis reconstruction with a simpler approach that uses the same information included in the training data, we performed an exhaustive search correlation: The BSP pattern to be reconstructed is correlated with all BSP patterns in the training data and the one with the maximum Pearson correlation coefficient is chosen. We assume that BSPs are already perfectly aligned to the time of excitation onset. In practice, precise alignment of the measured signals with the training data is difficult and therefore constitutes a major drawback of the approach. A time window of $T = \{20, 40, 80\}$ samples after excitation onset is then taken into account for correlation. This corresponds to different basis lengths L in the BSP basis reconstruction. Using MATLAB notation, the exhaustive search correlation can be described by:

$$i = \arg\max_i \operatorname{corr}\Big(\operatorname{reshape}\big(\mathbf{B}(:, 1{:}T), TM, 1\big),\ \operatorname{reshape}\big(\mathbf{B}_i(:, 1{:}T), TM, 1\big)\Big) \quad \text{with} \quad i = 1, 2, ..., 200$$

$\mathbf{B}$ are the measured BSPs and $\mathbf{B}_i$ are the BSPs of the i-th simulation in the training data. M is the number of electrodes. The localization results are shown in Fig. S1. It can be seen that the approach works well, if the CV matches the CV in the training data. For a CV of $0.8\,\mathrm{m/s}$, the results are best for $T = 40$. For a CV of 0.4 and $1.2\,\mathrm{m/s}$, mean errors are smallest for $T = 20$, at least for high SNRs. However, reducing T also decreases the robustness to noise. In conclusion, localization results for non-matching CVs are worse than with the BSP basis reconstruction, especially in terms of outliers.

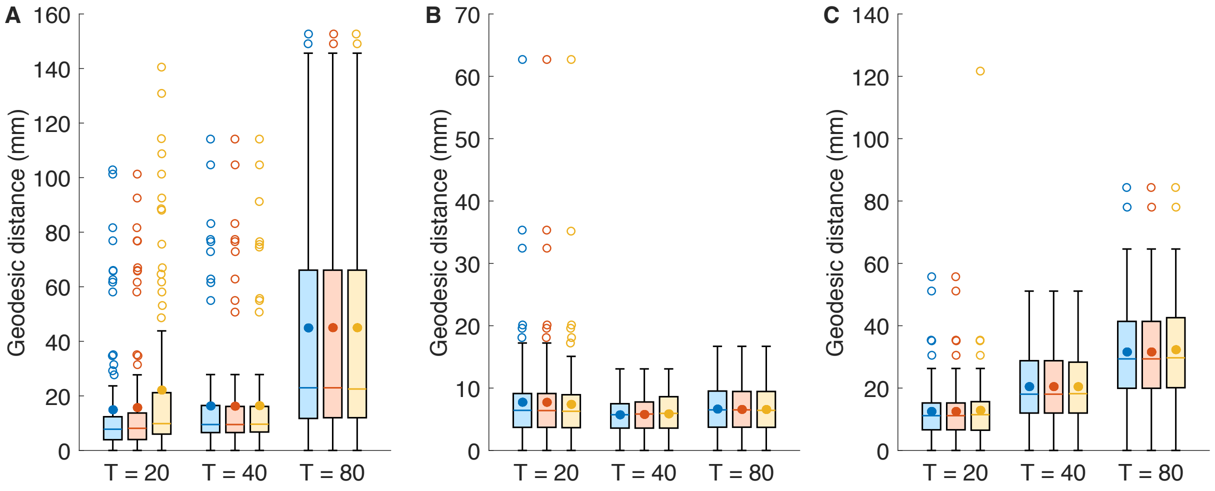

Figure S1. Localization errors for the exhaustive search correlation applied to all 100 ectopic foci. **A:** CV = $0.4\,\mathrm{m/s}$. **B:** CV = $0.8\,\mathrm{m/s}$. **C:** CV = $1.2\,\mathrm{m/s}$. Colors represent different SNRs. Blue: $40\,\mathrm{dB}$, red: $20\,\mathrm{dB}$, yellow: $0\,\mathrm{dB}$. Boxes: 25th to 75th percentile. Whiskers: 1.5 inter-quartile range. Filled circles represent the mean.

2 BSP BASIS RECONSTRUCTIONS FOR ANISOTROPIC ATRIA

In order to demonstrate that the BSP basis can also be used to reconstruct anisotropic spread of excitation, we created a highly anisotropic and heterogeneous model of the atria including the crista terminalis, pectinate muscles and Bachmann's bundle (Fig. S2). Fiber orientations were created using the rule-based approach described in [3]. Conductivities and anisotropy factors were defined as in [2]. Monodomain simulations for this anisotropic setup were computed on a tetrahedral mesh with 836 k nodes and an average edge length of 0.48 mm. The same forward calculation as for isotropic cases was used. Exemplary reconstructions were performed for four ectopic foci marked by black spheres in Fig. S2: Left inferior pulmonary vein, LA anterior-septal wall, RA lateral wall and RA appendage.
Fig. S3 depicts the reconstruction results for the anisotropic case in comparison to the isotropic case. Shown are the results for Tikhonov-Greensite (TikhGS) and a BSP basis of length $L = 33$. Although the reconstruction quality decreases compared to the isotropic case, the BSP basis reconstruction is still consistently better than TikhGS in terms of TMV and LAT correlation (spatial CC) and mean localization error (geodesic distance). The largest localization error occurs for the focus on the LA anterior-septal wall.

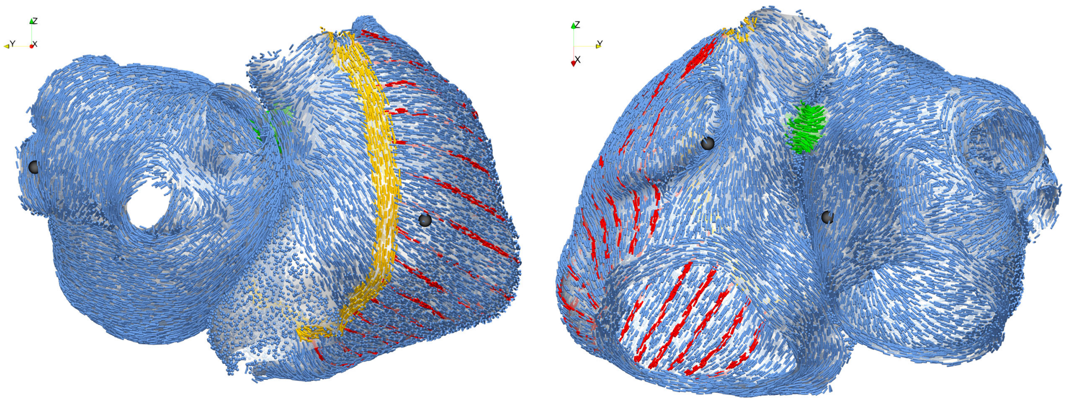

Figure S2. Anisotropic atrial geometry. Colors represent regions with different monodomain conductivities and anisotropy factors. Black spheres mark pacing locations.

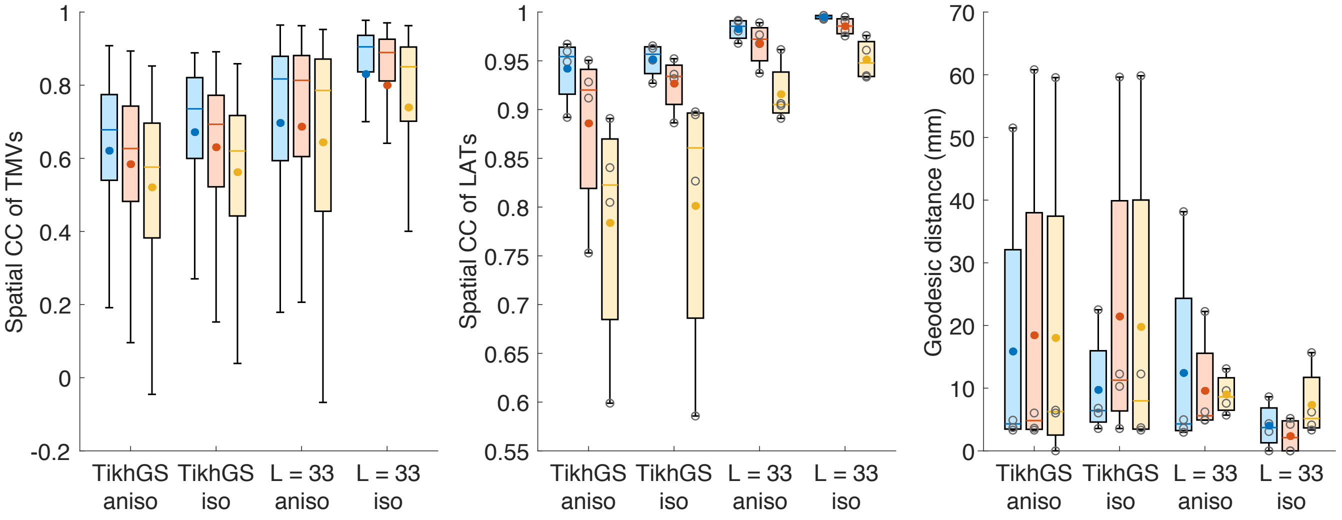

Figure S3. Reconstruction results of four ectopic foci for the anisotropic (aniso) and isotropic (iso) case. See Fig. S1 for a description of colors and markers. Grey circles represent individual ectopic foci. Box plots for the spatial CC of TMVs include all time steps individually and therefore no grey circles are shown.

3 BSP BASIS RECONSTRUCTIONS FOR SIMPLE ATRIAL FIBRILLATION

To show that the BSP basis generalizes from individual activities in the training data, reconstructions for the simulated case of simple atrial fibrillation from [1] were performed. The results for TikhGS and reconstructions using BSP bases of different lengths L are shown in Fig. S4. Although only focal activity was included to create basis vectors, they are still capable to coarsely approximate the large rotational activity on the right atrium. For low SNRs, reconstructions with large basis lengths perform better than TikhGS with respect to the mean spatial CC.

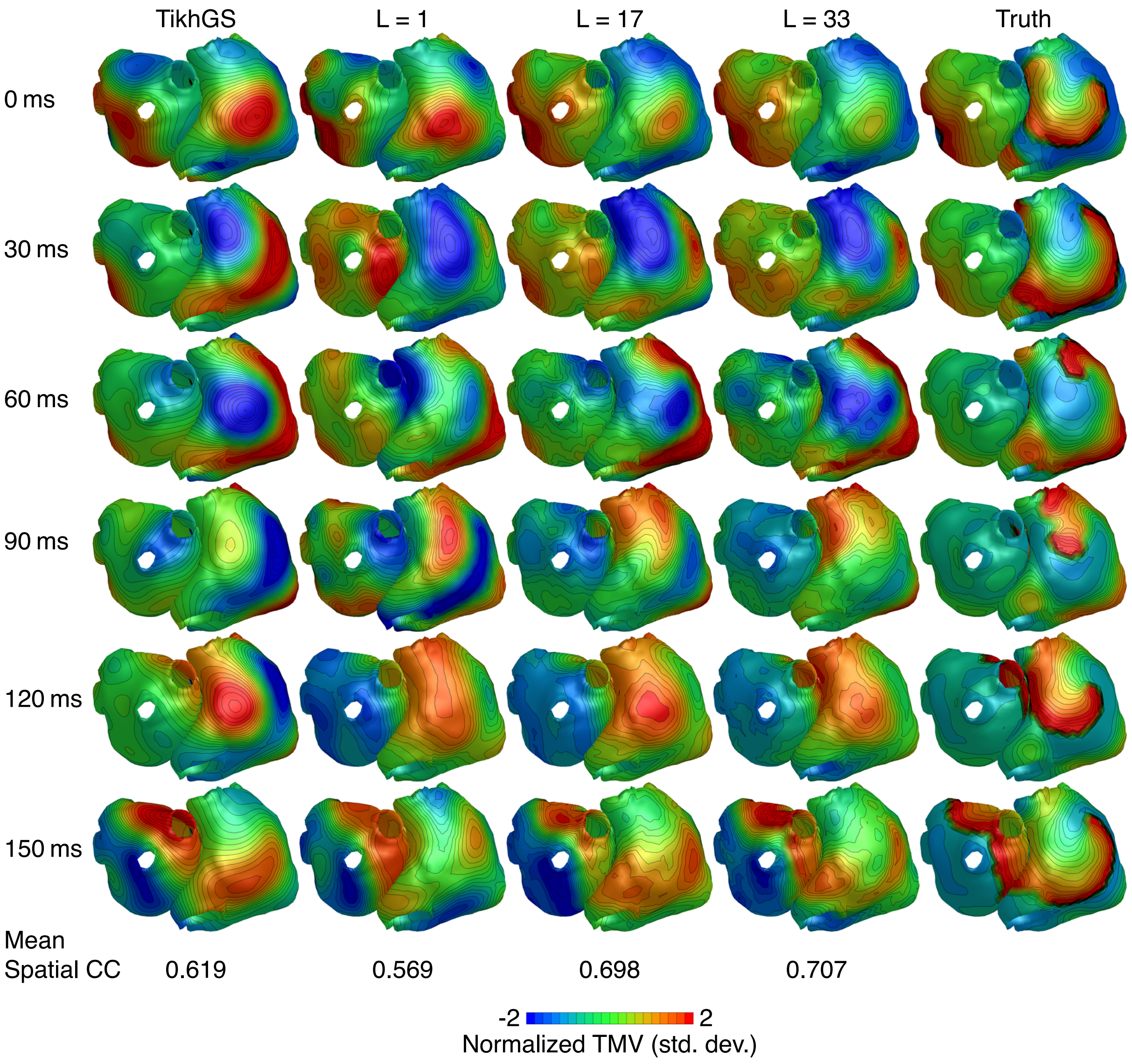

Figure S4. Reconstructions for a simulated case of simple atrial fibrillation and an SNR of $20\,\mathrm{dB}$. Transmembrane voltages (TMV) were normalized by subtracting the spatial mean and dividing by the spatial standard deviation.

REFERENCES

[1] Figuera, C., Suárez-Gutiérrez, V., Hernández-Romero, I., Rodrigo, M., Liberos, A., Atienza, F., et al. (2016). Regularization techniques for ECG imaging during atrial fibrillation: A computational study. *Frontiers in Physiology* 7, 466. doi:10.3389/fphys.2016.00466

[2] Loewe, A., Krueger, M. W., Platonov, P. G., Holmqvist, F., Dössel, O., and Seemann, G. (2015). Left and right atrial contribution to the p-wave in realistic computational models. In *International Conference on Functional Imaging and Modeling of the Heart* (Springer), 439–447

[3] Wachter, A., Loewe, A., Krueger, M. W., Dössel, O., and Seemann, G. (2015). Mesh structure-independent modeling of patient-specific atrial fiber orientation (De Gruyter), vol. 1, 409–412. doi:10.1515/cdbme-2015-0099
